# Hijama (wet cupping therapy) enhances oral and dental health by improving salivary secretion volume and pH in adult patients at King Abdul Aziz University Hospital (KAUH), Jeddah, KSA: A controlled trial study

**DOI:** 10.1016/j.jtumed.2022.07.012

**Published:** 2022-08-14

**Authors:** Fouzia A. Bukhary, Amal M. Obeid, Hanan M. Alsayyad, Ezzuddin A. Okmi

**Affiliations:** a Health Affairs Directorate, Ministry of Health, Makkah, KSA; b Yousef Abdulatif Jameel Scientific Chair of Prophetic Medicine Application, King Abdulaziz University Hospital, King Abdulaziz University, Jeddah, KSA; c King Abdulaziz University Faculty of Medicine, Jeddah, KSA; d Department of Communicable Diseases Prevention and Control, Saudi Public Health Authority, Riyadh, KSA

**Keywords:** حجامة, لعاب, غدة لعابية, تسوس أسنان, صحة الفم, Cupping, Dental caries, Hijama, Oral health, Saliva, Salivary gland

## Abstract

**Objective:**

The aim of this study was to explore the potential effect of Hijama in promoting oral health by analyzing its effects in modulating saliva flow and pH.

**Method:**

An open-label, non-randomized controlled trial design was conducted at the Hijama clinic of Y.A. Jameel Scientific Chair of Prophetic Medical Applications at King Abdul Aziz University Hospital (KAUH), Jeddah, KSA. Forty-one healthy volunteers were divided into two groups: Hijama (intervention, *N* = 21) and control (*N* = 20). Saliva volume and pH were measured in salivary samples collected in a standardized fashion, 1 h before admission to the Hijama room (pre-Hijama) and 30 min after the procedure (post-Hijama) in both groups. The Hijama group underwent an additional salivary collection 7 days after Hijama.

**Result:**

Early post-Hijama assessment showed an increase in saliva volume by an average of 1 mL in the Hijama group, whereas that in the control group decreased by 0.6 mL (*p* < 0.001; large effect size, Cohen's *d* = 1.24). Saliva pH also increased in the Hijama group by an average 0.22 but decreased by 0.08 in controls (*p* < 0.001; large effect size, Cohen's *d* = 1.22). The multivariate model demonstrated that Hijama explained 48.8% of the variability of both pH and volume together (group × time effect, eta squared = 0.488, *p* < 0.001), whereas time and sex had no effect. At 7 days post-Hijama, both the volume and pH of saliva had increased in the Hijama group with respect to the early post-Hijama time point; however, only the volume increase was statistically significant.

**Conclusion:**

Hijama enhanced salivary function and induced a significant increase in saliva volume and pH, which was maintained 7 days after the intervention. Further studies are warranted to identify other effects of Hijama on salivary glands and explore its long-term efficacy and clinical applications.

## الملخص

**أهداف البحث:**

كان الهدف هو استكشاف التأثير المحتمل للحجامة في تعزيز صحة الفم من خلال تحليل تأثيرها في تعديل تدفق اللعاب ودرجة الحموضة.

**طرق البحث:**

تم إجراء تجربة منضبطة غير معشاة ذات لصاقات مفتوحة في عيادة الحجامة في كرسي جميل العلمي للتطبيقات الطبية النبوية في مستشفى جامعة الملك عبد العزيز، جدة، المملكة العربية السعودية. تم تقسيم 41 متطوعا سليما إلى مجموعتين: مجموعة الحجامة (مجموعة تدخل، 21 متطوعا) والمجموعة الضابطة (20 متطوعا). تم قياس حجم اللعاب ودرجة الحموضة على عينات اللعاب التي تم جمعها بطريقة معيارية، قبل ساعة واحدة من الدخول في غرفة الحجامة (قبل الحجامة) وبعد 30 دقيقة (بعد الحجامة) لكلا المجموعتين. خضعت مجموعة الحجامة لعملية جمع لعاب إضافية بعد 7 أيام من الحجامة.

**النتائج:**

أظهر التقييم المبكر بعد الحجامة زيادة في حجم اللعاب بمتوسط 1 مل في مجموعة الحجامة مقابل انخفاض في المجموعة الضابطة بمقدار 0.6 مل. كما زادت درجة حموضة اللعاب في مجموعة الحجامة بمتوسط 0.22 ، بينما انخفضت بنسبة 0.08 في المجموعة الضابطة. أظهر النموذج متعدد المتغيرات أن الحجامة فسرت 48.8٪ من التباين في كل من الرقم الهيدروجيني وحجم اللعاب معا، بينما لم يكن للوقت والجنس أي تأثير. بعد 7 أيام من الحجامة، زاد حجم اللعاب ودرجة حموضته في مجموعة الحجامة مع الإشارة إلى وقت ما بعد الحجامة المبكر، غير أن فقط زيادة الحجم كانت ذات دلالة إحصائية.

**الاستنتاجات:**

عززت الحجامة وظيفة اللعاب وتسببت في زيادة حجم اللعاب ودرجة الحموضة التي تم الحفاظ عليها بعد 7 أيام من التدخل. هناك ما يبرر إجراء مزيد من الدراسات لتحديد الآثار النهائية الأخرى للحجامة على الغدد اللعابية واستكشاف فعاليتها على المدى الطويل والتطبيقات السريرية.

## Introduction

Saliva, the principal component of oral fluid, plays a critical role in the preservation of oral health, and the maintenance of oral homeostasis and microbiome balance, beyond other functions in facilitating food chewing and swallowing.^1^^,^^2^ Saliva is secreted by three pairs of major salivary glands: the parotid, submandibular, and sublingual glands. Moreover, it receives contributions from 300 to 400 minor salivary glands in the oral cavity.^3^^,^^4^ Human saliva in the oral cavity functions in the maintenance of human health, and its complex composition is indicative of normal or abnormal human health.^5^ Saliva flow and composition, as well as the percentage contribution of each gland, vary with physiological state, notably during mastication or food stimulation.^1^^,^^2^

Quantitative or qualitative changes in saliva have causal or syndromic relationships with several conditions, primarily oral diseases such as tooth decay and caries, wherein a variety of physical and biochemical changes in saliva have been documented.^6^, ^7^, ^8^, ^9^ However, salivary dysfunction may also be associated with or influenced by extra-oral conditions, such as menopause,^10^ aging,^11^ or radiotherapy,^12^ or by iatrogenic factors, such as treatment with isotretinoin,^13^ which may affect oral health. By contrast, saliva composition is influenced by a broad range of other physiological and pathological systemic conditions, such as nutritional status, substance use, and emotional, hormonal, or immunological statuses, in addition to several oncological and infectious diseases.^1^

Investigating saliva and its characteristics is an area of increasing interest among researchers and clinicians, because several saliva biomarkers have diagnostic and prognostic value and are easily accessible via noninvasive collection methods.^1^^,^^14^^,^^15^ Interventions modulating salivary flow or composition may have value in oral health preventive and therapeutic applications. For instance, several clinical trials have demonstrated that stimulating saliva production by chewing sugar-free gum has a protective effect against the development of dental caries.^16^ Likewise, the intake of tea, derived from *Camellia sinensis* dried leaves, has demonstrated a strong caries protective effect, owing to its antibacterial, amylase and acid production inhibitory, and fluoride supply properties.^17^ Consequently, saliva stimulation has been proposed as a preventive tool for promoting oral health by maintaining an optimal pH in the oral cavity.

However, some pathological conditions may skew saliva homeostasis toward a pro-caries state. For example, diabetes mellitus is associated with a decreased salivary pH, which is associated with a significantly elevated risk of dental caries and periodontitis among people with diabetes.^18^ The correlation of salivary flow or pH levels with dental caries development has been thoroughly demonstrated. An elevated pH, along with saliva buffering capacity and mineral content, is associated with decreased caries activity.^19^ By contrast, data from a 2-year longitudinal study have indicated that a low resting saliva pH (≤6.0) and flow (≤0.6 mL/min) are associated with a 60% and 140% increase in the incidence of dental caries.^20^ However, saliva properties change over time, thus potentially influencing the risk of active caries development in either direction.^21^

Hijama, wet cupping therapy, is a traditional remedy with a long history of use in several cultures and civilizations. In the Islamic tradition, the Prophet Mohammed, peace be upon him, has encouraged its use on several occasions, promoting it as one of the best remedies.^22^^,^^23^ During the past century, Hijama has regained popularity worldwide. Several clinical studies have been conducted to demonstrate its preventive and therapeutic effects in a variety of conditions and to adapt its technical aspects accordingly.^24^, ^25^, ^26^, ^27^, ^28^, ^29^, ^30^, ^31^, ^32^ Consequently, the practice of Hijama is regulated in several countries, notably in those in which this practice has high popularity, such as KSA, which has developed national standards of safety and training.^22^ Other studies have identified the mechanisms of action of Hijama, including enhancement of local blood circulation, tissue clearance of oxidative stress and inflammatory mediators, and immunomodulatory effects.^31^^,^^33^, ^34^, ^35^ However, studies on the effects of Hijama on oral health and dental health are scant. In KSA, few studies have evaluated the effects of wet cupping on saliva. Therefore, this study fills this research gap by increasing knowledge on this topic. Moreover, if effective and sustained effects of Hijama in stimulating saliva are demonstrated, this treatment may provide a better preventive option that is cost-effective. Furthermore, this study may aid in exploring the potential effects of Hijama in promoting oral health and preventing dental caries by analyzing the modulation of salivary gland function. The aim of this study was to assess the effects of Hijama on saliva by measuring the changes in saliva flow and pH after a single Hijama session performed at two time intervals among adults attending the Prophetic Medicine Clinic of Y.A. Jameel Scientific Chair of Prophetic Medical Applications at King Abdul Aziz University Hospital (KAUH), Jeddah, KSA.

## Materials and Methods

### Design and setting

This is an open-label, non-randomized controlled trial design performed at the Prophetic Medicine Clinic of Y.A. Jameel Scientific Chair of Prophetic Medical Applications at KAUH, Jeddah, KSA, from March 31, 2019 to January 12, 2020. The KAUH Prophetic Medicine Clinic is part of the outpatient clinic department in KAUH. It is funded by Y.A. Jameel and accepts referrals from different specialties in the university hospital. Cupping therapy is performed as a complementary therapy for different conditions in conjunction with routine treatment. The benefit of cupping therapy is systematically assessed by comparison of the outcomes of the routine treatment method combined with cupping therapy. On average, 2000 patients are seen in the cupping therapy clinics every year. The clinic has three qualified physicians and three qualified nurses. The physicians and nurses are licensed by the Saudi Commission for Health Specialties and for cupping therapy by the National Saudi Organization of Integrative Medicine.

### Participants

The study included apparently healthy adult patients who attended the Hijama Clinic for preventive and health promotive purposes. Individuals who had a clinically detectable oral condition, such as tooth decay, aphthous lesions, gingivitis, or labial herpes, or who had undergone dentistry or an oral procedure in the prior 3 months were excluded. Likewise, individuals with uncontrolled chronic diseases, such as hypertension, diabetes, dysthyroidism, end-stage disease, ongoing malignant disease, pregnancy, or mental disorders, were excluded.

Participants were divided into two groups: an intervention group (Hijama group) and control group. The group allocation was determined according to participant preference. Participants from each group received a full explanation of the study and signed a consent form for participation in the study as volunteers.

### Intervention

Participants from the intervention group were seated on the examination bed at a 45° angle, with the head and neck resting against the back of the bed. Four cups were applied to each patient. The cups were located at the parotid and submandibular salivary gland areas bilaterally. Parotid cups were placed at the parotid area just anterior to the tragus of the ear bilaterally. Submandibular gland cups were placed just medial to the midpoint of the ramus of the mandible, also bilaterally. Standard sterilized, single-use commercial Hijama sets were used. Sets included cups equipped with a vacuum system and a suction pump. Practitioners with sterile gloves placed the vacuum cups, which were suctioned onto each identified point of the skin (dry cupping). Each cup was maintained for 30 s and was removed by de-suction. Afterwards, a size 15 surgical sterile scalpel was used to make small, light, superficial cuts of 1 mm depth and 1.5 mm length in the circular area to be covered by the cup. The cups were then repositioned, mild suction was exerted, and the cups were kept under suction for 2 min. This procedure was repeated two or three times. The cups were then removed, and their fluid content was disposed of in a biological waste container. Importantly, the cuts did not bleed except after suction was exerted; the released blood-like fluid was filtered out through the Hijama suction, because the cuts were too superficial to cause any bleeding. On the cut skin points, a simple sterile dressing was placed. The duration of the complete session was approximately 15–20 min.

### Control

Participants were Hijama clinic attendees who agreed to provide two salivary samples for our study. The salivary samples were collected through the same technique described below for both the control and interventional groups.

### Saliva collection and outcomes

The present study focused on two outcomes, saliva flow and pH, which were assessed in pre- and post-Hijama salivary samples. The changes in saliva volume and pH from baseline to post-intervention was measured twice. The immediate effect was measured from baseline (30 min before the start of the Hijama session) to 30 min to 1 h post-intervention. Then the delayed effect was measured from baseline to 7 days post-intervention.

Salivary samples were collected with standard measuring cups used to collect biological fluid samples. Saliva was collected for all participants in a straight sitting position; participants were instructed to spit into the cup on demand for a 5-min duration, without any stimulation (non-stimulating saliva secretion).^36^ In the intervention group, pre-Hijama saliva collection was performed 30 min before the start of the Hijama session, whereas post-Hijama collection in another cup was performed through the same method, 30 min to 1 h after the cupping session. In the control group, saliva was collected twice, at a 1-h interval, with the same method, into two cups.

After documentation of the saliva volume in cc, the saliva pH was measured with a Pocket Pen Water pH Meter Digital Tester PH-009 (ASIN: B07MQL6X5T), according to the manufacturer's guidelines.

A second post-Hijama saliva collection for flow and pH measurement was performed for the intervention group 7 days after the Hijama session to measure delayed effects. The control group underwent a single outcome measurement, as specified previously. All saliva collections followed the same procedure described previously.

### Statistical methods

Statistical analysis was performed with the Statistical Package for Social Sciences (SPSS) version 21.0 for Windows (SPSS Inc., Chicago, IL, USA). Categorical variables are presented as frequency and percentage, whereas continuous variables are presented as mean ± standard deviation (SD). Intergroup analysis compared the control and intervention groups' regarding pre- and post-intervention assessments, as well as pre-to-post intervention changes, with both parametric (independent *t*-test) and nonparametric (Mann–Whitney U test) tests. In intragroup analysis, Wilcoxon signed-rank test was used to compare the pre- versus post-intervention saliva volume and pH within each group, separately. The effect size of the intervention was estimated with Cohen's *d* coefficient. Repeated-measures (RM) ANOVA was used to analyze the effects of group, time, and time × group on outcomes (saliva pH and volume). Results are presented as lambda Wilk's or Pillai's trace statistics, as appropriate, and the calculated squared eta indicated the percentage of variability in the outcome accounted for by each factor. A squared eta ≥0.14 was assumed for a large factor effect. Paired *t*-test was used to analyze the changes in pH and volume from pre- to post-intervention in each group separately. A *p* value < 0.05 was considered to reject the null hypothesis.

## Results

### Baseline group characteristics

Forty-one participants were enrolled: 21 in the intervention group (all women) and 20 in the control group (16 women; *p* = 0.048). The mean (SD) baseline saliva volume and saliva pH were 3.35 (1.13) mL and 6.82 (0.49), respectively, and no statistically significant difference was observed between the intervention and control groups (*p* > 0.05; Table 1).

**Table 1:** Baseline characteristics (*N* = 41).

Parameter	Category	Total	Control (*N* = 20)	Hijama (*N* = 21)	*p*-value
Sex	Male	4 (9.8)	4 (20.0)	0 (0.0)	
	Female	37 (90.2)	16 (80.0)	21 (100.0)	0.048∗^a^
Saliva pH	Unit, mean (SD)	6.82 (0.49)	6.70 (0.56)	6.94 (0.38)	0.103
Saliva volume	mL, mean (SD)	3.35 (1.13)	3.38 (1.13)	3.33 (1.15)	0.908

∗ Statistically significant result (*p* < 0.05).

a Fisher's exact test

### Short-term effect of Hijama on saliva flow

**Figure 1:**
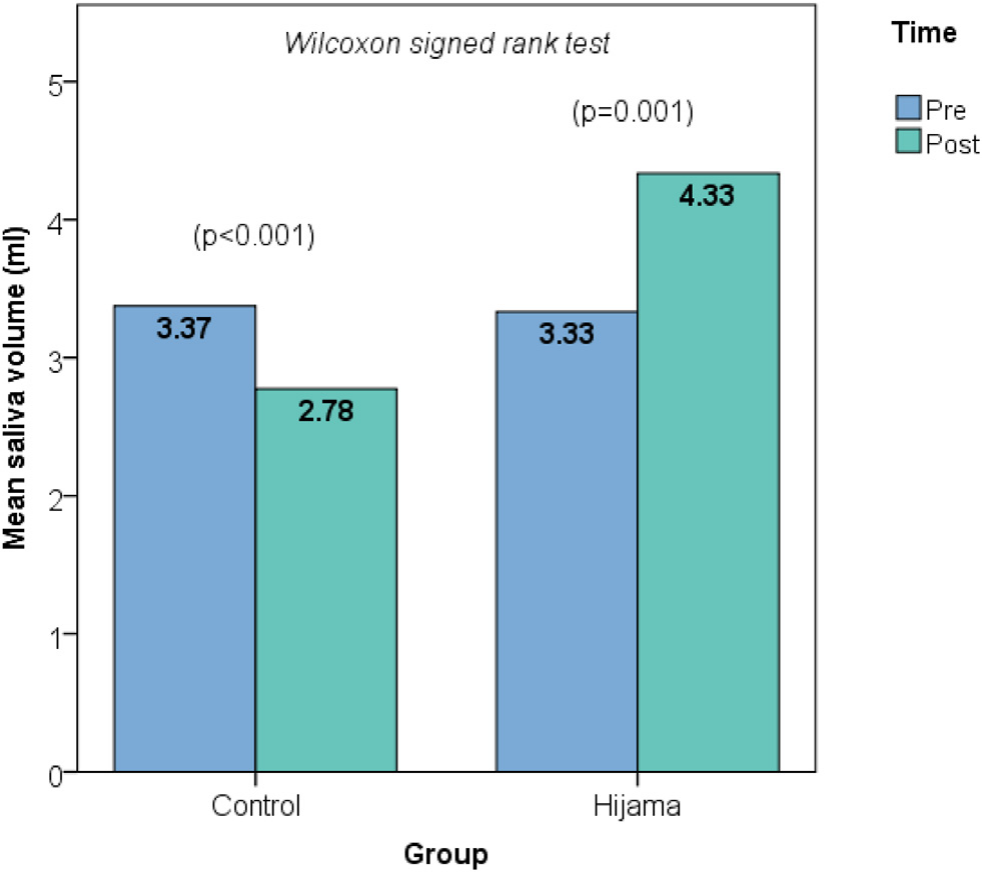
Changes in saliva volume from pre- to post-intervention in the Hijama and control group (paired analysis): the mean saliva volume from pre- to post-intervention increased significantly in the Hijama group but decreased in the control group.

An intergroup comparison of the pre-to-post intervention change in saliva volume showed an increase in the Hijama group (mean change = 1.00 mL) but a decrease in the control group (mean change = −0.60 mL); the difference was statistically significant (*p* < 0.001; Table 2). Intragroup paired analysis with Wilcoxon signed-ranked test showed that both the increase in the Hijama group (*p* = 0.001) and the decrease in the control group (*p* < 0.001) in saliva volume were statistically significant (Figure 1). The effect size of the intervention was large (Cohen's *d* = 1.24).

**Table 2:** Short-term effect of Hijama on saliva volume (intergroup comparison; *N* = 41).

Parameter	Category	Control (*N* = 20) mean SD		Hijama (*N* = 21) Mean SD		*p*-value^1^	*p*-value^2^
Post intervention volume	mL, mean (SD)	2.78	1.26	4.33	1.24	<0.001∗	0.001∗
Δ volume	mL, mean (SD)	−0.60	0.48	1.00	1.18	<0.001∗	<0.001∗

Δ volume: pre to post (30 min after intervention) change in saliva volume. ∗ Statistically significant result (*p* < 0.05).

Test used: ^1^ independent *t*-test, ^2^ Mann Whitney U test.

### Short-term effect of Hijama on saliva pH

**Figure 2:**
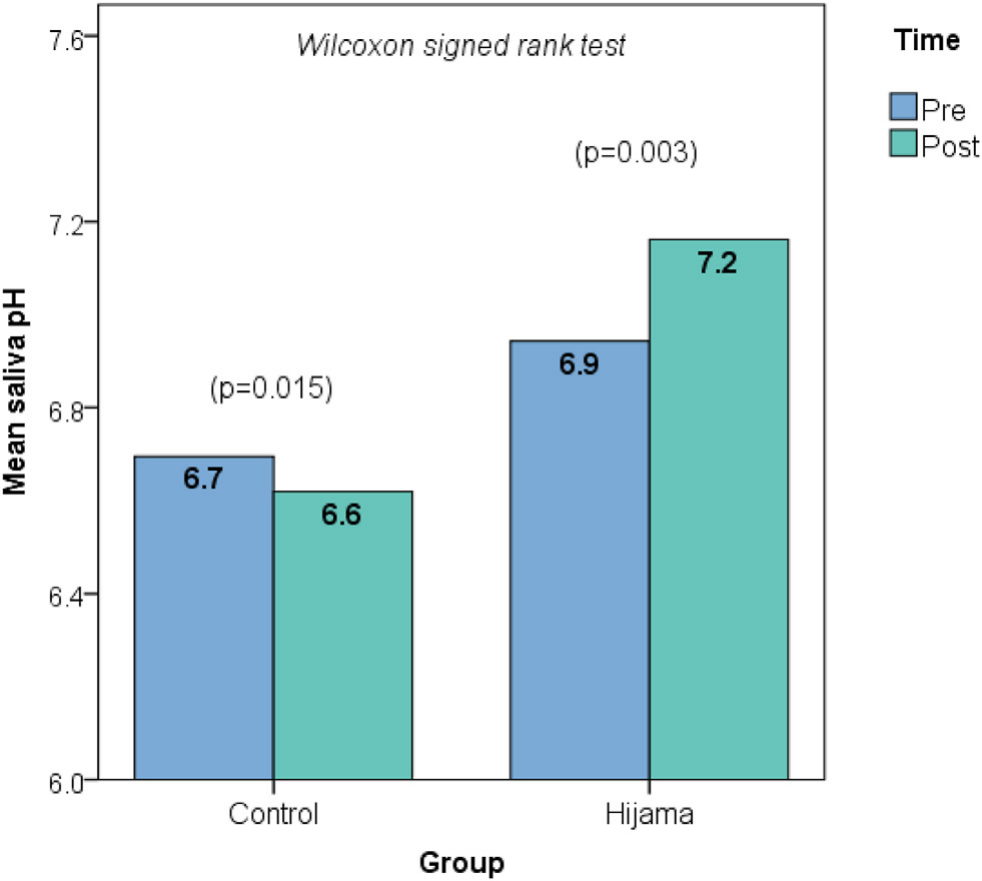
Changes in saliva pH from pre- to post-intervention in the Hijama and control groups (paired analysis): mean saliva pH from pre- to post-intervention increased significantly in the Hijama group but decreased in the control group.

An intergroup comparison of the pre-to-post intervention change in saliva pH showed an increase by 0.22 in the Hijama group versus a decrease by 0.08 in the control group; the difference was statistically significant (*p* < 0.001; Table 3). Intragroup paired analysis showed that both the increase in the Hijama group (*p* = 0.003) and the decrease in the control group (*p* = 0.015) in saliva pH were statistically significant (Figure 2). The effect size of the intervention was large (Cohen's *d* = 1.22).

**Table 3:** Effect of Hijama on saliva pH (intergroup comparison).

Parameter	Category	Control (*N* = 20)		Hijama (*N* = 21)		*p*-value^1^	*p*-value^2^
Post intervention pH	Unit, mean (SD)	6.62	0.51	7.16	0.36	<0.001∗	0.001∗
Δ pH	Unit, mean (SD)	−0.08	0.13	+0.22	0.30	<0.001∗	<0.001∗

Δ pH: pre to post (30 min after intervention) change in saliva pH. ∗ Statistically significant result (*p* < 0.05).

Test used: ^1^ independent *t*-test, ^2^ Mann Whitney U test.

### Short-term effect of Hijama on saliva pH and volume: RM ANOVA

**Figure 3:**
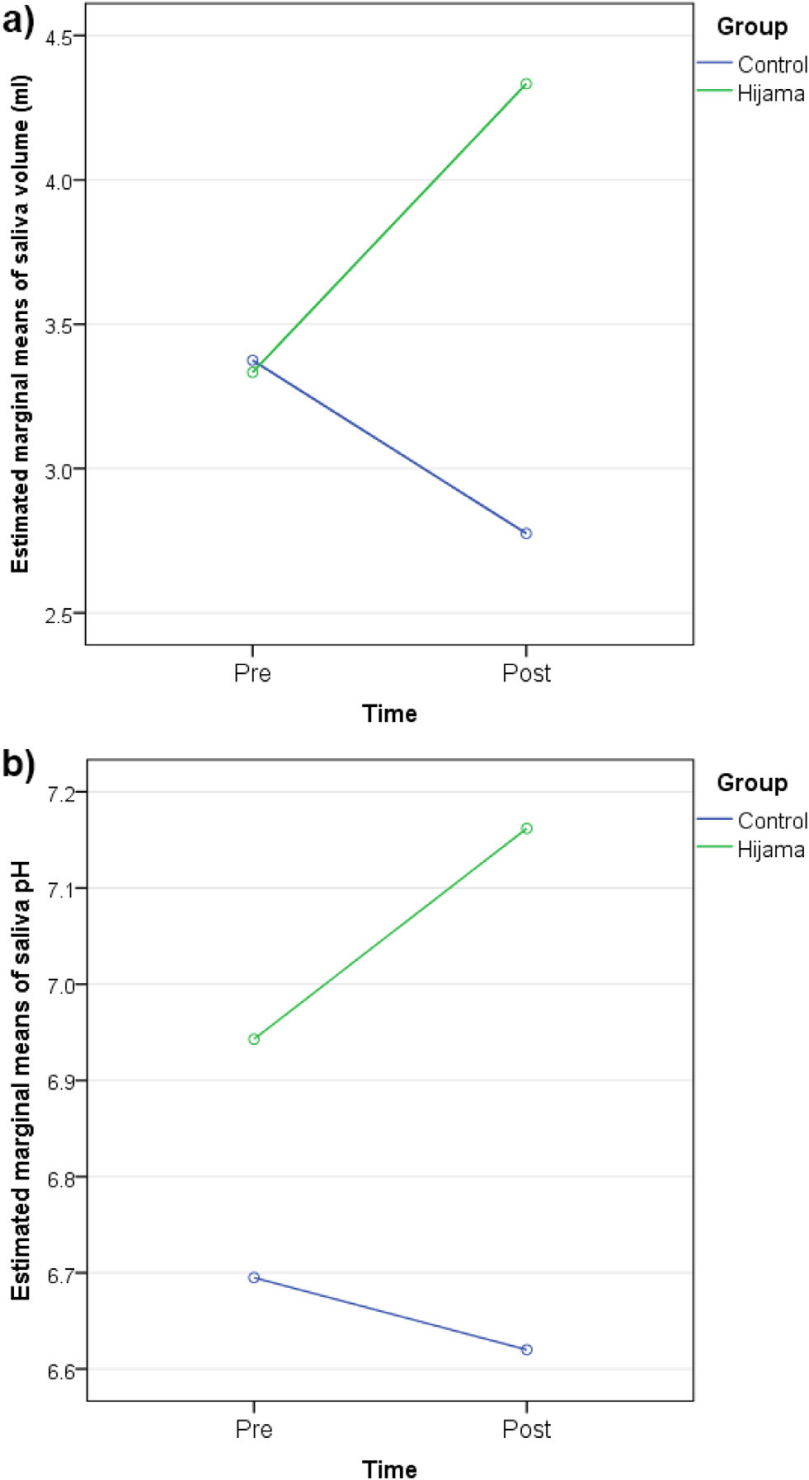
**(a), (b**). Effect of Hijama on saliva volume and pH (RM ANOVA). Figures show the pre- to post- intervention changes in the estimated marginal means of saliva volume (a) and pH (b) in the Hijama versus control groups.

Hijama explained 44.8% of the variance in saliva volume (group × time effect, eta squared = 0.448, *p* < 0.001) and 29.6% of the variance in saliva pH (group × time effect, eta squared = 0.296, *p* < 0.001) from pre- to post-intervention (30 min after the intervention). The multivariate model demonstrated that Hijama explained 48.8% of the variability of both pH and volume together (group × time effect, eta squared = 0.488, *p* < 0.001). All three models showed no effect of time alone in explaining the variance in saliva volume or pH (*p* > 0.05; Table 4). The estimated marginal means of saliva volume and pH are depicted in Figure 3, showing diverging curves for both outcomes between the Hijama and the control group.

**Table 4:** Effect of Hijama on saliva pH and volume (RM ANOVA).

Outcome/factor	Lambda Wilk's/Pillal's trace^a^	*p*-value	Squared eta^c^	Interpretation
**Volume**				
Time	0.048	0.167	0.048	No effect
Group × time effect	0.448	<0.001^b^	0.448	Very large effect
**pH**				
Time	0.092	0.055	0.092	No effect
Group × time effect	0.296	<0.001^b^	0.296	Large effect
**Multivariate model**				
Group (inter-subject)	0.807	0.017^b^	0.193	Large effect
Time	0.895	0.122	0.105	No effect
Group × time effect (intra-subject)	0.512	<0.001^b^	0.488	Very large effect

a Lambda Wilk's statistic was used if Levene's equality of error variance test was verified; otherwise, Pillal's trace statistics were used.

b Statistically significant result (*p* < 0.05).

c Represents the percentage of variability in the outcome that is accounted for by the factor; the effect was considered large for squared eta ≥ 0.14.

### Sex-specific responses to Hijama

Another RM ANOVA multivariate model including sex as a cofactor showed no effect for sex alone (eta squared = 0.009 [*p* = 0.552] and 0.000 [*p* = 0.970]), sex × group (eta squared = 0.000 and 0.000, *p* values not calculable), sex × time (eta squared = 0.003 [*p* = 0.718] and 0.001 [*p* = 0.814]), or sex × time × group (eta squared = 0.000 and 0.000, *p* values not calculable) in the change in saliva volume and pH from pre- to post-intervention, respectively (**results not presented in tables**).

### Delayed effects of Hijama on saliva volume and pH

**Figure 4:**
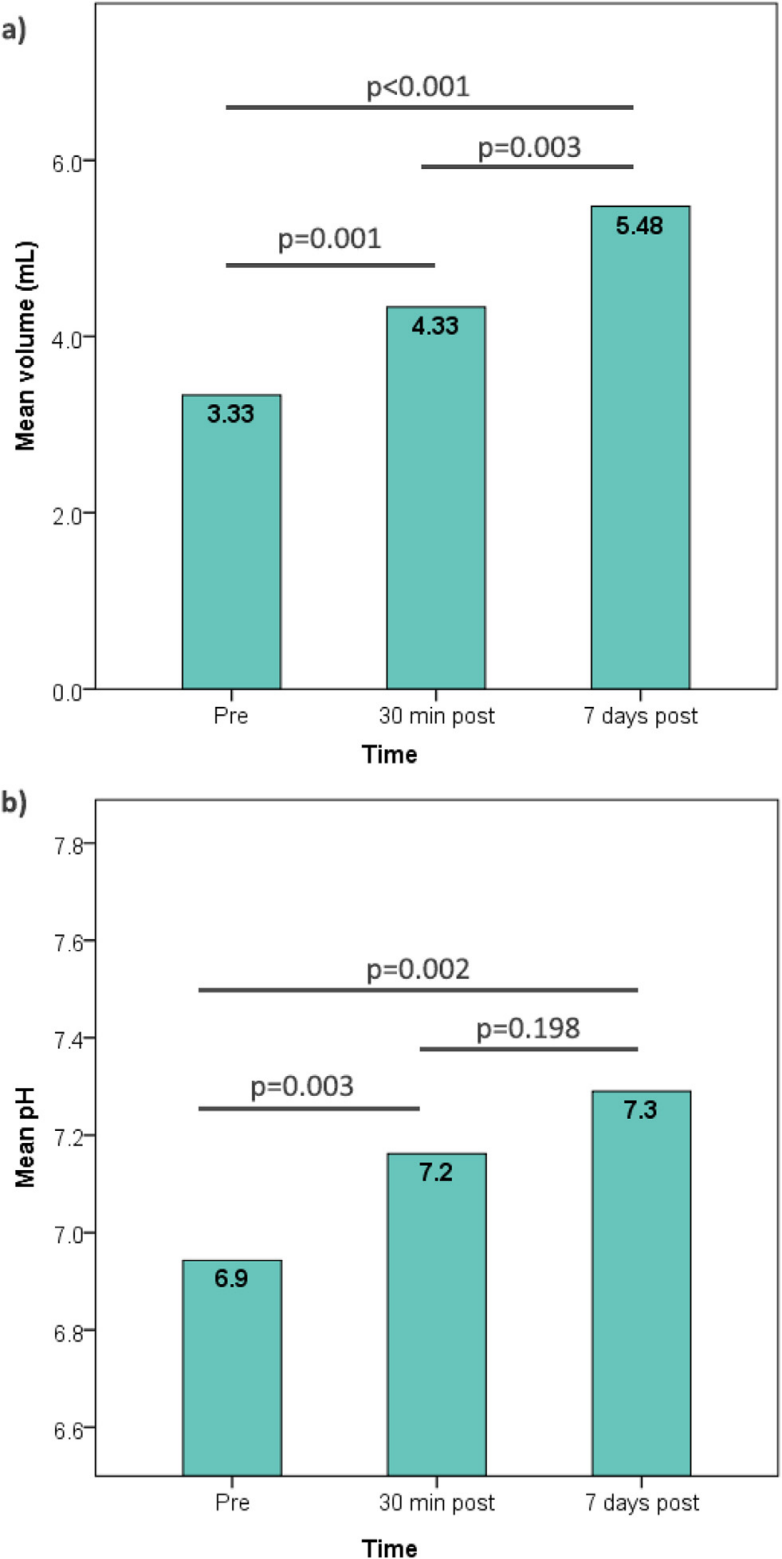
**(a), (b**) Progression of salivary volume and pH in early (30 min) and delayed (7 days) post-Hijama assessments in the intervention group. Figures show a continual increase in saliva volume (a) and pH (b) 7 days after a single Hijama session. The levels of statistical significance (*p*-values) are calculated between two time points with paired *t*-tests.

The 7 day post-intervention assessments of the Hijama group showed a mean saliva volume of 5.48 mL, a value significantly higher than those in the pre-intervention (*p* < 0.001) and early post-intervention assessments (*p* = 0.003). By contrast, although saliva pH further increased at 7 days post-intervention (mean = 7.29), the difference was significant with respect to only the pre-intervention assessment (*p* = 0.002) but not the early post-intervention assessment (*p* = 0.198; Figure 4).

## Discussion

Our study was aimed at measuring the effects of wet cupping on saliva volume and pH. This study may be the first to investigate the effects of Hijama in dental and oral health. The findings demonstrated that cupping increased the saliva volume and pH. Moreover, the difference in sex distribution between the Hijama and control groups did not affect the observed increase in saliva volume and pH in the Hijama group compared with the control group. Additionally, the baseline characteristics showed comparable saliva volumes and pH between study groups, despite the significant difference in sex distribution. The potential effect of sex is discussed in the following section.

This study indicated that Hijama induced an early and large-effect increase in both saliva and pH. Investigation of the mechanisms underlying these effects on saliva was not within the scope of the present study and should be the objective of further studies. However, the literature has suggested several mechanisms of actions for Hijama, including interference with saliva stimulation and the enhancement of local microcirculation with vasodilatory effects, thus facilitating draining and immediate elimination of noxious materials and toxins from interstitial compartments. Additionally, Hijama has been demonstrated to increase blood flow, thus stimulating the autonomic nervous system.^37^ A study in middle school students with multiple caries has indicated a high prevalence of autonomic dysfunction associated with hyperactivation of the sympathetic nervous system.^38^ Another study in patients with type 1 diabetes has indicated an association between impaired saliva secretion and autonomic nervous system dysfunction.^39^ Hijama has also been demonstrated to decrease oxidative stress by removing oxidative molecules such as myeloperoxidase.^40^ Several studies have demonstrated a positive association between caries, or the risk of caries development, and the levels of oxidative markers in both the saliva and serum.^41^^,^^42^ Another study has suggested a role of the clearance of microparticles, also called extracellular vesicles, which are released by aging erythrocytes, platelets, endothelial cells, or leukocytes, and have been associated with pro-inflammatory states and thrombophilia profiles.^43^ However, as previously stated, further studies are warranted to explore the mechanisms underlying the observed effects of Hijama on saliva flow and pH, as well as other effects requiring further investigation.

The aim of the present trial was to explore the effect of Hijama in inducing positive changes in saliva flow and pH that might help prevent the development of caries. The effect of saliva stimulation on dental caries prevention was investigated and demonstrated several decades ago.^18^, ^19^, ^20^ Simulating saliva enhances its clearance, buffering power, and degree of saturation with inorganic components such as calcium and phosphate. Additionally, stimulated saliva has high concentrations of bicarbonate. The combination of these effects results in two principal pH-raising mechanisms: clearance of dietary carbohydrates from the oral cavity and buffering of plaque acidity, in addition to the enhancement of tooth remineralization.^20^^,^^44^ These observations led to several clinical trials exploring the effects of saliva stimulation in decreasing the incidence of dental caries. A review including seven clinical trials has reported that chewing sorbitol-containing chewing gum after each meal is associated with a 6.4%–39% decrease in the 2- to 3-year risk of caries development. The preventive effect was significant only with strict use of chewing gum after each meal three times per day; otherwise, the beneficial effect was not significant.^45^ This constraint that may limit the clinical use of chewing gum. By contrast, our study showed that one session of Hijama may have promising preventive effects against dental caries by increasing saliva volume and pH.

Sex-specific differences in saliva characteristics are expected, on the basis of physiological differences, particularly the effects of sexual hormones such as estrogen on the salivary glands. However, the literature is inconsistent regarding the presence of sex differences in saliva flow or pH, or other biochemical components such as α-amylase.^46^^,^^47^ A study by Pandey et al. has estimated the sex-specific differences in saliva flow rate and pH among school-aged children. The authors divided the study population into two age groups of 7–10 years and 11–15 years. In the 7–10 year group, the mean (SD) saliva flow was 0.310 (0.10) versus 0.299 (0.12) mL/min (*p* = 0.787), and the mean (SD) pH was 7.17 (0.52) versus 7.15 (0.76; *p* = 0.934) in caries-free boys versus girls, respectively. In the age group of 11–15 years, the mean (SD) saliva flow was 0.302 (0.08) versus 0.278 (0.07) mL/min (*p* = 0.389), and the mean (SD) pH was 7.01 (0.68) versus 7.03 (0.58; *p* = 0.932) in caries-free boys versus girls, respectively.^46^ By calculating the *p* values, we observed that none of the abovementioned differences were statistically significant, thus not supporting sex-specific differences in unstimulated saliva flow and pH. Nonetheless, some authors have reported lower saliva flow rates among women than men, in both unstimulated and stimulated saliva.^48^^,^^49^ Other studies have shown sex-specific differences in other biochemical components of saliva, thus theoretically leading to baseline differences in pH. For example, Bel'skaya et al. have shown that saliva calcium and urea concentrations are higher in men than women; however, only the age groups of 40–49 and 50–59 years showed a significant difference for calcium and urea, respectively. Similar differences in uric acid concentration were observed in all age groups; however, none were statistically significant.^50^ By focusing on post-stimulation changes, as in the present study, a study by Liu-Hui has demonstrated that, although saliva volume and pH increased in both sexes after citric acid stimulation, the values remained significantly lower in women than men.^51^ This finding further supports the conclusion that the increase in saliva volume and pH observed in the present study was unlikely to have been due to sex differences between the intervention and control group but instead were attributable to Hijama.

### Limitations

The generalizability of the present study findings is limited by the small sample size, the non-randomized enrollment of patients, and the high risk of a placebo effect, because of the impracticability of blinding, and the high subjective and emotional implications of Hijama in the target population's culture.

The sample size of male participants was small, because the group allocation was determined according to participant preference. No male participants preferred to be included in the intervention group.

Although observations from the 7th day post-Hijama support a sustained effect of Hijama in enhancing saliva flow, both quantitatively and qualitatively, this finding was not controlled, given the lack of 7-day assessment of the untreated group.

## Conclusion

The results of this study showed that wet cupping resulted in greater saliva flow and pH shortly after and 7 days after a single Hijama session than that in the control group with no intervention. The effects of Hijama in increasing the saliva volume and pH in preventing dental caries are promising, and support potential clinical applications of Hijama in oral health promotion. Our studies showed that Hijama enhanced salivary function and induced a significant increase in saliva volume and pH that was maintained 7 days after intervention. However, further studies are warranted to identify other effects of Hijama on dental health and to explore the underlying mechanisms. Further controlled studies with larger sample sizes and longer follow-up times are warranted to demonstrate these effects and their influence on the incidence of dental caries by providing a better preventive option that is cost-effective.

## Source of funding

This research did not receive any specific grant from funding agencies in the public, commercial, or profit sectors.

## Conflict of interest

The authors have no conflict of interest to declare.

## Ethical approval

Ethical approval was granted for the study by the IRB committee of research department, Health affairs of Makkah region, Ministry of Health (IRB #: H-02-K-076-0419-114). Ethical approval date:02/05/2019.

## Authors contributions

FAB provided research materials, and participated in the literature review, methods, and discussion. AMO conceived and designed the study, conducted research, provided research materials, wrote the initial and final drafts of the article, provided logistic support, and participated in the methods and discussion. HMA collected, organized, analyzed, and interpreted data; and participated in participated in the methods and discussion. EAO participated in the literature review and discussion, provided logistic support, organized data, and reviewed the final draft of the article. All authors have critically reviewed and approved the final draft and are responsible for the content and similarity index of the manuscript.
